# Treatment of Ulcers of the Leg by Spender’s Chalk Ointment and Tight Bandage

**Published:** 1854-03

**Authors:** Henry S. Patterson


### *Treatment of Ulcers of the Leg by Spender's Chalk Ointment and Tight Bandage.* By Henry S. Patterson, M. D.

While a resident pupil of our Alms House Hospital, in 1836, I received and read the book (then recently published) of Mr. Spender, of Bath, on Ulcerous Diseases of the Leg. Notwithstanding its great prolixity and wearisome repetitions, (faults due to the writer's attempt to make a book from material about sufficient for a moderate journal article,) I was much interested in the direct common sense of its practical directions. The "sore legs" being regarded as a surgical bore, and left entirely to the care of the *internes*, I resolved to test Mr. S.'s practice. The winter of 1836–7 being very severe, ample opportunity was presented. In the white men's surgical ward and "long garret" we had 125 chronic non-specific ulcers of the leg, besides others among the negroes and women. A majority of these were put under the use of the chalk ointment and tight bandage, the dressing being attended to, as far as possible, by myself and colleague personally. The result was entirely satisfactory. A subsequent opportunity of observation in the same institution, as well as considerable experience in private practice, has convinced me of the decided superiority of this method over any other in general use. I have therefore thought it would be useful to recall professional attention to a surgical authority who, never attracting much notice, appears now to be undeservedly forgotten.

The treatment recommended by Spender is based on the conviction that the obstinate and unmanageable character of ulcers of the leg arises, in the great majority of cases, from a varicose condition of the veins of the limb. Not only where the larger venous trunks are so dilated as to be conspicuous at first sight, but also in instances where inspection is required to perceive the lesion, does he regard the varix as the source of all difficulty. In many cases, an œdematous condition of the limb, or adventitious deposites of a more solid character, may mask entirely the enlargement of vessels. In other cases, minute examination and especially the touch will reveal an enlarged and nodulous condition of many of the smaller superficial veins, although the main trunks are not yet varicose. The consequence will be an impeded and imperfect circulation, and impaired vitality of the tissues of the limb. Hence come the liability to ulceration and the difficulty of healing an ulcer, when once formed. Mr. Spender found this state of things in about 80 per cent. of the sore legs coming under his care. I am inclined to consider this estimate short of the truth. I have never seen an ulcer of the leg (non-specific) of long standing, unaccompanied by a varicose condition of the adjacent veins. Obviously, the first step in the treatment of such ulcer, is to remedy this primary morbid condition.

All surgeons recognize the "varicose ulcer" as a species. The peculiarity of Mr. S. consists in asserting its frequency. The necessity of correcting the diseased condition of the vessels, when existing, is perceived by all. Two methods of meeting this indication are proposed. The first, which is that recommended by most writers, consists of rest in the recumbent posture. This is always inconvenient and objectionable, and often impossible. The other plan is to maintain firm and uniform compression of the entire limb from toe to knee. Such is the practice of Mr. S., and he carries it out by the tight bandage. The adhesive strips as recommended by Mr. Baynton, and so generally used, cannot accomplish this purpose. They do good by local compression of the surface of the sore, and by approximating the edges, so as to obtain a smaller surface of cicatrix, but they do not act like the roller. Another advantage of the latter is, that the patient may not only move about with it on, but will be actually benefitted by the exercise. Mr. S. was in the habit of directing his patients to take even more than the ordinary exercise. The tissues, moreover, that are thus formed, are much more likely to be permanent than parts repaired in bed with the limb elevated. It is, unfortunately, too common for legs, healed by rest, to begin to slough and erode as soon as the patient returns to his ordinary avocations.

Mr. S. next endeavors to ascertain what is the natural process of cure in an ulcer, with a view to imitate that process as closely as possible. He finds it to consist in an incrustation or scabbing, first of the edges and then of the whole sore, and the formation of a cicatrix under the protection of this crust. But upon this subject, and upon the abuse of local applications, we must let him speak for himself.

"If a beast in the field stakes itself, the blood pours out, and some of it coagulating, forms a crust on the wounded part. If the injury is not very extensive, this covering may probably be sufficient to protect it from all external influence, and to allow the formation of a healthy skin underneath, when the cure will be very speedily and effectually performed. Even the union by the first intention, as it is called, seems in some degree to be indebted to the presence of the blood acting on this principle. At other times, when the accident is more severe, suppuration takes place, and a purulent secretion is thrown out, which, in a short period, becomes dry, forming a scab first on the margin and then on the surface of the sore. This shields the part from all outward injury, and thus covered and protected, a new skin is formed, and the crust drops off. If by any means the scab be removed before the cure is completed, another quantity of fluid is supplied, which hardens in the same manner as the former, but then all the intermediate time is lost. Although, from our habits of interference, we do not possess the same opportunities of seeing the natural process of cure in the human subject, yet it may be occasionally observed. Besides the manner in which most slight scratches and cuts are spontaneously healed, in what is commonly termed "breaking out" on the lips, every one knows that if the scab be picked off too soon, the sore becomes exposed, another scab is formed, and it is only by letting it alone that the part underneath will heal. Now if we compare this beautiful, simple, and effective process with the method of treating wounds and ulcers on the human subject by poultices and fomentations, vapors and lotions, together with spungings, washings, and wipings, how striking the difference appears; and it is really wonderful that we can continue to go on pursuing a plan which so violates these first lessons of nature. We must become more humble as well as more observant, and be contented to abandon our complicated and cumbersome remedies, which by losing their simplicity, not uncommonly lose their effect, before we can hope to make much advancement in the treatment of diseases. Nature is to be conquered only by submitting to her; and he conquers the most easily who submits the most readily." Pages 62 and 63.

"This application, (the poultice) in most instances, acts prejudicially, likewise, by the effects which it produces on the shape and character of the sore. It makes the granulations soft, pale, and flabby; and converts the margins into spongy, insensible edges, giving them a sharp and abrupt termination, the most unfavorable conditions imaginable for healing. It also absorbs the purulent fluid which is formed on its surface, preparatory to the act of reparation. It has been clearly proved, that an ulcerated part, deprived of its matter, unless covered and protected by a scab, is arrested in its progress of healing, and does not proceed until the fluid is reproduced. It is true that if one supply of the pus is taken away, another is thrown out, but then the intermediate space is lost. Besides, the renewal of the poultice is each time a repetition of the evil, as every successive one tends to absorb the matter that is present, and the surface becomes continually deprived of its fluid almost as fast as it is formed. Thus, then, both the unhealthiness of the parts below, and the nature of the sore above, require the very opposite treatment to poulticing , and it is really difficult to assign any other cause for the frequent and indiscriminate use of such a remedy, than the disinclination to do anything which will soil the fingers or offend the nose. We can order a poultice, and even prescribe a fomentation or a lotion, and yet stand at a very agreeable distance—shifting off on other hands the local treatment of the disease, instead of executing it by our own. It is, however, very clear, that if the performance of what ought to be done strikes us with disgust, though it may be allowable to dislike it, yet if the aversion prevents us from doing our duty, we are inexcusable for pretending to undertake the management of the case at all." p. 93.

Our author cannot say too much in condemnation of the poultice in these cases. It is astonishing how constantly patients apply to one even yet with their legs enveloped in that irritating abomination, a sour bread-and-milk poultice. By surgeons they are much less used than formerly, but still far too frequently. To ulcers of the leg they should never be applied, except to cleanse them when sloughing. The only poultice I have used for years is the simple paste of ground flaxseed, or "cake-meal," and boiling water. To this may be added a little Liq. Sodæ Chlorin., to overcome fœtor, or powdered charcoal, or yeast, as a corrective to the sore. With regard to the preparation of the edge in very old sores, I do not think that Mr. S. is sufficiently explicit. Mere compression will not answer in very many cases. In some cases there is a mass of unseparated cuticular deposit which, after softening by a poultice, may be gently separated by the handle of the scalpel. But in others the cutis itself is thickened, callous, and semi-cartilaginous, often inverted and the ulcer burrowing beneath. The only remedy is its removal, without which cicatrization will not commence. This may be affected by the caustic alkali or the knife. Nitrate of silver will not answer, often making no impression whatever on the indurated mass. The scalpel, however, is always preferable, and should be used freely so as to secure a new and healthy edge. This done, the next thing is to procure incrustation, which Mr. S. proposes to effect by his chalk ointment, the main peculiarity of his practice. His object in this ointment is to have a bland, unirritating, impalpable powder, held together by the smallest quantity of unctuous matter that will permit its being spread and otherwise managed. He employs prepared chalk and fresh lard, in the proportion of three or even four parts of the former to one of the latter. The formula which he prefers, and which I have generally adopted, is as follows:—

| | |
|---|---|
| Take of Prepared Chalk, | 4 lbs. |
| Fresh Lard, | 1 lb. |
| Olive Oil, | 3 oz. |

To the lard and oil, melted together, the chalk should be added gradually, being first rubbed to a fine powder and passed through a sieve. The mass should then be stirred until nearly cold. It may be rendered still smoother by subsequent rubbing in a marble or wedgewood mortar, but this will scarcely be required. It is impossible to prepare the ointment properly by mere trituration without the aid of heat, as I have seen apothecaries attempt. Under all circumstances there will be a slight degree of grittiness, due to the chalk, but not enough to constitute a practical objection. In private practice, however, I have used an ointment prepared with the precipitated carbonate of lime, which is perfectly smooth. The comparative costliness of this substance would be an objection to its adoption in hospital practice, or where, from the size of the sore or the frequency of dressing, the quantity used is very great.

The ointment is spread, about the thickness of a wafer, upon linen or cotton cloth, and applied over the whole sore, extending some distance beyond its margin. The next step is to apply a tight bandage from the toe to the knee. Mr. S. recommends a roller of calico (muslin) or flannel, two inches wide and six or eight yards long. The flannel he employs only in old and feeble subjects, or where there is much œdema of the limb. The bandage should be applied firmly and smoothly *by the surgeon himself.* No man can possibly apply a bandage properly to his own limb, and very few nurses, even in Hospitals, will be found competent to this duty. As the success of the treatment depends very much upon the manner in which the bandage is applied, the only safe plan is for the surgeon to attend to it himself. It should be made to embrace the limb closely, and may be drawn as tight as possible. Occasional reverse turns or folds will be necessary, and it should be fastened firmly with two or three circular turns below the knee. Properly applied it will remain for days and even a week or more. The longer the sore is left undisturbed the better. It should not be opened unless there is pain or fœtor, or the discharge is sufficient to soil the dressing offensively. At first, the rapid reduction in the size of the limb, from the absorption of serous effusion and even of more solid adventitious deposit, will cause the bandages to slacken and require their more frequent renewal. In many cases, however, I have changed the dressing only once in a week or ten days, and with the best results. The laced stocking I have used in a few cases, as a substitute for the bandage, but it did not answer my purpose. The patient complained much more of inconvenience, and there was a want of uniform compression about the ankle, where it is particularly needed. The elastic stocking of vulcanized caoutchouc, or *bas contre les varices*, made by Vié, of Paris, and now on sale here, I have employed in several instances with the most satisfactory results. It answers admirably as a preventive of ulcer in varicose limbs, or as a protection after the sore is healed, but it cannot become a substitute for the bandage in the treatment of the sore.

When this dressing is removed the surface will be found almost universally improved, clean and free from offensive odor. The discharge would seem to be in a great measure absorbed, and its acrimony neutralized by the chalk. At the same time it will be perceived that a thin layer of chalk has been deposited on the edges of the sore, and sometimes also in patches in the middle. *This must not be disturbed on any account*, as beneath it is forming the tender new cicatrix. Neither must the limb be wiped or washed. If wet with discharge, it may be dried by a soft cloth gently pressed upon it. A new dressing of the ointment should be then laid upon it, and the bandage reapplied as quickly as possible. With each removal of the dressing the crust will be seen to have encroached still farther upon the surface, until it is finally covered. If found to crack and become irregular, its separation may be assisted gently. The applications (especially the bandage) should be continued for some time after the ulcer is entirely cicatrized.

This treatment I can from extended experience confidently recommend to my professional brethren. It is applicable to all ulcers of the leg in which a varicose condition of the superficial veins constitutes the element of difficulty and delay in the cure, and these will be found (I am satisfied) to be four-fifths of all the cases that occur. Mr. Spender entirely disregarded the usual distinction of ulcers into the indolent and irritable, treating both alike. Some of the very worst irritable sores I have ever seen have yielded at once to the chalk ointment and compression. A recent case illustrates this point. An elderly gentleman applied to me with a very painful "pimple," as he termed it, which had afflicted his leg then for a couple of weeks, and would not heal. I found the lower third of the limb considerably swollen, and, over the sharp edge of the tibia, a prominence, on the summit of which was a superficial ulcer, about a line in diameter, covered with an orange-yellow sloughy matter, and discharging a transparent yellow ichor, streaked with blood. It was attended with constant and intense burning pain, and the surrounding parts were deep red and tender to the touch. All the mucilaginous poultices, &c. heretofore used, had only appeared to make it worse. Being actively engaged in business, the patient declared rest utterly out of the question. Here was a fair case of commencing irritable ulcer, under unfavorable circumstances. The large venous trunks were not varicose, but the smaller ones were prominent and nodulated to the touch. I applied the chalk ointment and an elastic stocking, and the pain was relieved instantly.

The chalk was continued for two weeks, when the sore was entirely healed and soundly cicatrized, but the stocking was worn for several months.

